# Melatonin feeding changed the microbial diversity and metabolism of the broiler cecum

**DOI:** 10.3389/fmicb.2024.1422272

**Published:** 2024-08-19

**Authors:** Li Zhen, Yi Huang, Xuewen Bi, Anyu Gao, Linlin Peng, Yong Chen

**Affiliations:** ^1^College of Animal Science and Veterinary Medicine, Heilongjiang Bayi Agricultural University, Daqing, China; ^2^Key Laboratory of Bovine Disease Control in Northeast China, Ministry of Agriculture and Rural Affairs, Daqing, China; ^3^Heilongjiang Provincial Key Laboratory of Prevention and Control of Bovine Diseases, Daqing, China

**Keywords:** melatonin, broiler, cecum, intestinal contents, microorganisms

## Abstract

To study the effect of melatonin supplementation on the gut microbes of broilers, 160 healthy 3-week-old Ross 308 broilers with similar body weights were selected and randomly divided into four groups (M0, M20, M40, and M80) supplemented with 0, 20, 40, or 80 mg/kg melatonin. The results showed that the abundance-based coverage estimator (ACE) index of cecum microorganisms was significantly lower in the M80 group. The dominant phyla of intestinal contents in the M0, M20, M40, and M80 groups were Bacteroidetes and Firmicutes. The M40 group showed an increase in the relative abundance of Bacteroidetes spp. in the intestine, while the relative abundance of Ruminococcus spp. in the intestine of the M20, M40, and M80 groups was significantly greater than that of the M0 group. Kyoto Encyclopedia of Genes and Genomes (KEGG) functional analyses revealed that the supplementation of melatonin increases the expression of genes related to cellular processes (cell motility, cell growth and death, and cellular community-eukaryotes), environmental information processing (membrane transport and signal transduction), and genetic information processing (transport and transcription), and Cluster of Orthologous Groups (COG) of proteins functional analyses revealed that the supplementation of melatonin resulted in a significant increase in cellular processes and signaling (cell motility, signal transduction mechanisms, intracellular trafficking, secretion, and vesicular transport), information storage and processing (RNA processing and modification, chromatin structure and dynamics, translation, ribosomal structure, and biogenesis), metabolism (energy production and conversion, lipid transportation and metabolism, inorganic ion transport and metabolism, secondary metabolite biosynthesis, transport, and catabolism), and poorly characterized (general function prediction only). In summary, supplementation of feed with melatonin can increase the diversity of intestinal microorganisms and the relative abundance of Bacteroides and Firmicutes in the cecum, improve digestive ability and nutrient absorption ability, and positively regulate the metabolic ability of broilers.

## 1 Introduction

Melatonin, an indole-like hormone secreted by the pineal gland in animals, has a variety of physiological functions such as regulation of biological rhythms (Zisapel, 2018; Vasey et al., 2021), antioxidants (Tan et al., 2015; Reiter et al., 2017), and immunomodulation (Carrillo-Vico et al., 2013; Muñoz-Jurado et al., 2022). Since its first discovery in the 1950s, melatonin has been studied continuously, covering a wide range of fields from neurophysiology to immunology and from human medicine to animal science.

Melatonin is distributed in the pineal gland, retina, intestines, and parabasal lacrimal glands, as well as in the gastrointestinal tract of mammals and birds (Reppert et al., 1995). Melatonin concentrations in the gastrointestinal tract are 10–400 times higher than those in the blood (Acuña-Castroviejo et al., 2014; Gong et al., 2022). Melatonin secretion in the gut is controlled by diet rather than circadian rhythms (Challet, 2019). Melatonin in the gastrointestinal tract is mainly produced by intestinal chromaffin cells, certain types of immune cells, and intestinal commensal cells. Studies have shown that melatonin can improve intestinal health through various mechanisms, including regulating the mammal intestinal peristalsis (Song et al., 2005; Chen et al., 2011), enhancing the mouse intestinal barrier function (He et al., 2020; Ma et al., 2020), and inhibiting the mouse intestinal inflammatory responses (Wang et al., 2022; Zhang B. et al., 2022; Zhang L. et al., 2022). In addition, melatonin has a powerful antioxidant effect, which can scavenge free radicals of chronic intermittent hypoxia-induced intestinal barrier dysfunction and reduce the damage of oxidative stress on intestinal cells, thus protecting the intestinal mucosa (Li et al., 2023).

The diversity of gut microbiota plays an important role in host metabolism, nutrient digestion, growth performance, and health (Brown et al., 2023). The chicken cecum, the most important intestinal portion of the distal part of the chicken gut, is a complex ecosystem of highly diverse microbiota, an important part of the digestive system of poultry, and the most active area of microbial activity (Lyte et al., 2022; Koçak and Özaydın, 2023). Its microbial community plays a key role in nutrient metabolism, immunomodulation, and disease prevention and control. Cecum microorganisms not only participate in the fermentation and metabolism of feeds to produce beneficial metabolites such as short-chain fatty acids (SCFAs) but are also able to inhibit the growth of pathogenic bacteria through competitive exclusion and the production of antimicrobial substances. Microorganisms in the cecum can promote animal growth by converting fibrous components into digestible components through fermentation (Cisek et al., 2023). Appendectomy greatly reduces the rate of metabolism of crude fat or other nutrients in poultry compared to normal poultry (Chaplin, 1989). The diversity and stability of the cecum microbiota are closely related to the growth performance and health status of broilers. Analyzing the cecum microbiota is a key area of poultry nutrition research and helps to understand the diversity of cecal bacteria and their interactions with the host (Li et al., 2017; Yin et al., 2023).

Studies have shown that the anti-inflammatory functions of melatonin in the gut are mediated by inhibiting the production of proinflammatory cytokines such as TNF-α, IL-6, and IL-1β; improving intestinal permeability; and decreasing the levels of proinflammatory cytokines and chemokines, which can attenuate organ inflammation and alter the intestinal microbiota in both animals and humans (Zhang B. et al., 2022; Zhang L. et al., 2022). Moreover, melatonin reversed osteolysis-induced dysbiosis and increased the relative abundance of microorganisms that produce SCFAs and the expression of butyrate synthase. The mechanism by which melatonin attenuates titanium particle-induced osteolysis through the enrichment of the gut microbiota metabolite butyrate is due to melatonin’s effective inhibition of butyrate enrichment-mediated activation of NOD-, LRR-, and pyrin domain-containing protein 3 (NLRP3) inflammatory vesicles (Yuan et al., 2018; Arioz et al., 2021). In addition, melatonin reverses inflammatory osteolysis-induced dysbiosis and increases the relative abundance of SCFA-producing bacteria. In addition, melatonin reverses inflammatory osteolysis-induced dysbiosis and increases the relative abundance of SCFA-producing bacteria (Wu et al., 2021).

Melatonin alters the composition of the intestinal microbiota (Park et al., 2020), increases the abundance of the phyla Thicket and Bacteroidetes, and decreases the proportion of harmful bacteria in mice (Bai et al., 2019). At the same time, gut microbes modulate the synthesis of their melatonin precursors, tryptophan and 5-hydroxytryptamine (5-HT), and regulate melatonin secretion. Tryptophan availability can be caused by changes in the diet or gut microbiota structure. The intestinal microbiota regulates three tryptophan metabolic pathways. In the indole pathway, the microbiota characteristics determine the type of indole derivatives, and these indole analogs are effective in the intestinal mucosa, immune system, and other areas. In addition, Toll-like receptors activated by inflammatory factors and pathogen-associated molecules secreted by the intestinal microbiota activate the kynurenine pathway. SCFAs in the gut promote 5-HT secretion from intestinal chromaffin cells and melatonin secretion. Spore-producing bacteria in the intestine can activate intestinal chromaffin cells by secreting other metabolites that promote colonic 5-HT production (Yi et al., 2023).

Although the potential of melatonin in regulating gut health has been initially revealed, studies on its specific effects on cecum microbial diversity in broiler chickens are still relatively limited. This study aimed to investigate the impact of different levels of melatonin on the microbial diversity of broiler cecum and provide a new theoretical basis and practical guidance for poultry feeding management and gut health maintenance.

## 2 Materials and methods

### 2.1 Experimental design and animal management

A total of 160 3-week-old Ross broilers were selected and randomly divided into four treatment groups, with eight replicates in each group and five birds in each replicate, supplemented with 0, 20, 40, or 80 mg/kg melatonin in standard broiler diets (mixed before feeding).

The broilers were fed commercially available broiler feed. They were fed a 0–21 day feed (2% crude protein, 1% calcium, 0.6% phosphorus, and 0.5% methionine) in the early period (up to 3 weeks of age). In the later period (4–5 weeks of age), the broilers were fed late feed (20% crude protein, 0.9% calcium, 0.5% phosphorus, and 0.5% methionine). The broilers were kept in three-layer cages with the temperature regulated by warming lamps, with *ad libitum* feeding and light hours from 5:50 a.m. to 9:50 p.m. during the 21-day experiment. All experimental protocols were approved by the Animal Care and Use Committee on Animal Ethics of Heilongjiang Bayi Agricultural University.

### 2.2 Sample collection and processing of cecum contents

On day 22 of the experiment, four Ross broilers were randomly selected from each group. After weighing, the bird’s cervical vertebrae were dislocated and the cecal contents were removed, dispensed into 1.5 mL tubes, immediately snap-frozen with liquid nitrogen for 15 min, and stored in a −80°C freezer.

### 2.3 Analysis of the microbial diversity of cecal contents

The DNA was extracted with the TGuide S96 DNA Kit [Tiangen Biotech (Beijing) Co., Ltd.] according to instructions. The DNA concentration was measured with the Qubit HS Assay Kit (Invitrogen, Thermo Fisher Scientific, OR, USA). The universal primer set was used to amplify the V3–V4 region of the 16S rRNA gene. The process of PCR was performed in a total reaction volume of 10 μL: DNA template 5–50 ng, Vn F (5′-ACTCCTACGGGAGGCAGCA-3′) (10 μM) 0.3 μL, Vn R (5′-GGACTACHVGGGTWTCTAAT-3′) (10 μM) 0.3 μL, KOD FX Neo Buffer 5 μL, dNTP (2 mM each) 2 μL, KOD FX Neo 0.2 μL, ddH2O up to 10 μL. After the individual quantification step, amplicons were pooled in equal amounts. For the constructed library, Illumina NovaSeq 6000 (Illumina, Santiago, CA, USA) for sequencing was used. The data obtained by sequencing on the Illumina NovaSeq platform and FLASH v1.2.7 software were used to merge the reads through overlap, and the obtained merged sequences were the raw tags. The spliced raw tags were filtered to obtain high-quality tag data, and the chimeric sequences were identified and removed using UCHIME v4.2 to obtain the final effective tags. The tags were clustered at the 97% similarity level using Usearch software, and the OTUs were taxonomically annotated based on the Silva (bacteria) and UNITE (fungi) taxonomic databases. Based on the results of the OTU analysis at each taxonomic level, the community composition of each sample was obtained at the taxonomic levels of kingdom, phylum, order, family, genus, and species. Mothur (version v.1.30) and QIIME software were used for alpha diversity analysis and beta diversity index difference analysis. The alpha diversity index was obtained by using the Ace, Chao1, and Shannon indices and sample dilution curves. PICRUSt was used to predict the functions of microbial communities.

### 2.4 Statistical analysis

The *p*-value was obtained from t-tests of the species abundance data between groups using Metastats, and the *p*-value was corrected to obtain the *q*-value. Finally, species causing differences in the composition of the two groups were screened based on *p*-values (or *q*-values) with a default *p*-value of ≥0.05. One-way analysis of variance (ANOVA) and t-tests were performed using GraphPad Prism 9 to compare the differences in α diversity and key microbiota between groups. A *p*-value of <0.1 was almost clinically significant. A *p*-value of <0.05 was considered to indicate statistical significance.

## 3 Results

### 3.1 Effect of dietary melatonin supplementation on the OTUs of cecum microorganisms

**Figure 1:**
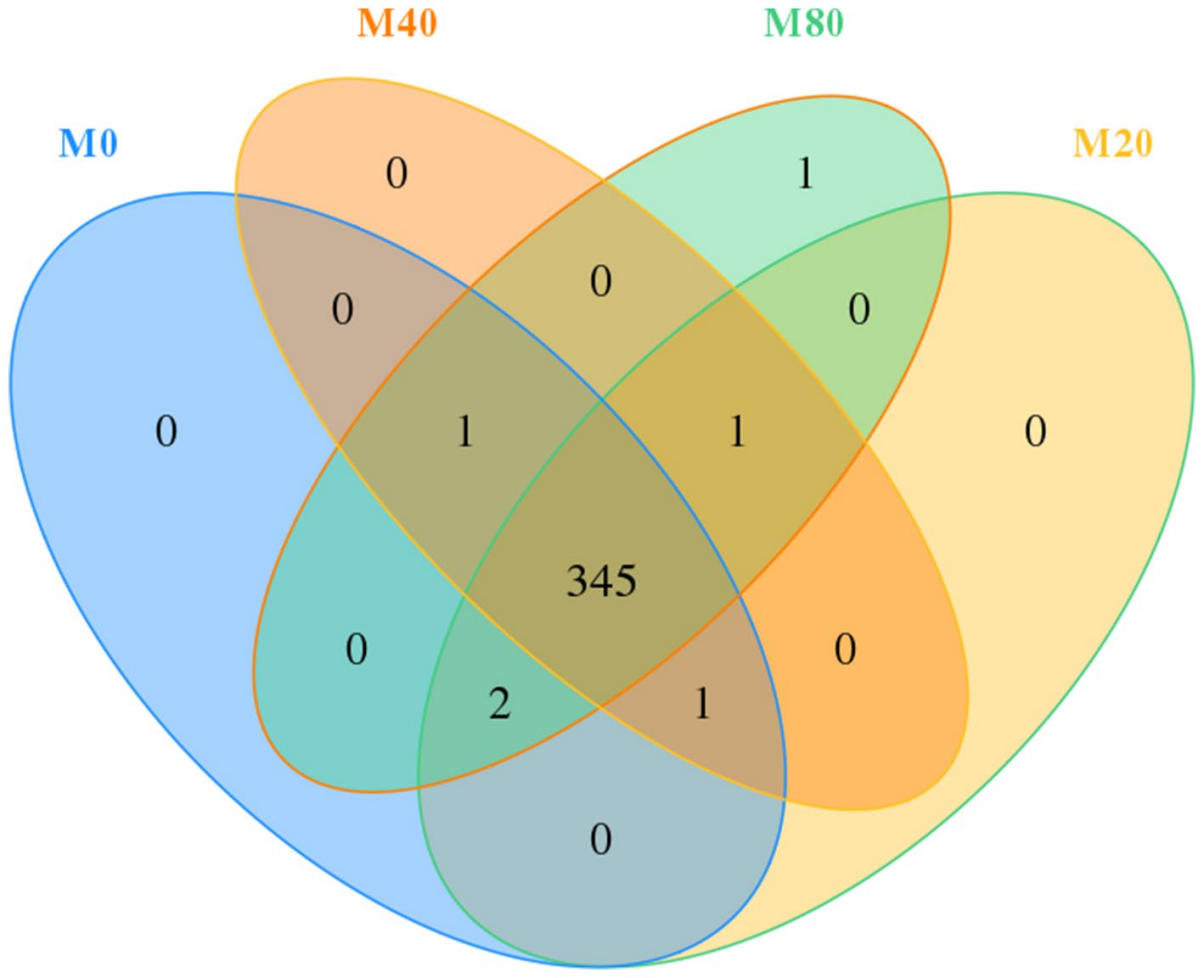
Venn diagram of the distribution of microbial OTUs in the cecum of broilers supplemented with melatonin.

Usearch software was used to cluster tags at a 97% similarity level, obtain OTUs, and taxonomically annotate OTUs based on the Silva and UNITE taxonomy databases to obtain the number of OTU samples. A total of 1,396 OTUs were obtained from the cecum contents of broilers in the four groups. The total number of OTUs shared by the four groups was 345, of which groups M0, M20, M40, and M80 contained 349, 349, 348, and 350 OTUs, respectively, and groups M0, M20, M40, and M80 contained 0, 0, 0, and 1 unique OTU, respectively (Figure 1).

### 3.2 Supplementation with melatonin alters the community composition of the gut microbiota

**Figure 2:**
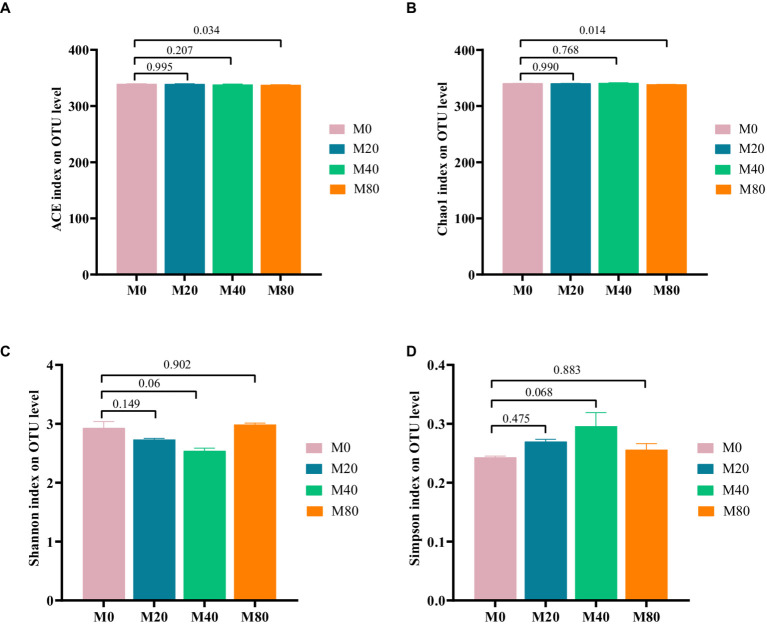
Alpha diversity analysis of cecum microorganisms in melatonin-supplemented broiler diets. **(A)** ACE index, **(B)** Chao1 index, **(C)** Shannon index, and **(D)** Simpson index.

Microbial alpha diversity indices in the cecum of the broilers in each group were analyzed based on OTU classification levels. The ACE index (Figure 2A) and the Chao1 index (Figure 2B) of cecal microorganisms were significantly lower (*p* < 0.05) in the M80 group than in the M0 group, and there was a trend toward a lower Shannon index (Figure 2C) and Simpson index (Figure 2D) in the M40 group (*p* < 0.1). There were no significant differences in the ACE index (Figure 2A), Chao1 index (Figure 2B), Shannon index (Figure 2C), or Simpson index (Figure 2D) of the cecal microorganisms in the M20 groups compared to those in the M0 group, or the ACE index (Figure 2A), Chao1 index (Figure 2B) of the cecal microorganisms in the M40 group, or the Shannon index (Figure 2C), Simpson index (Figure 2D) of the cecal microorganisms in the M80 group compared to those in the M0 group (*p* > 0.05).

**Figure 3:**
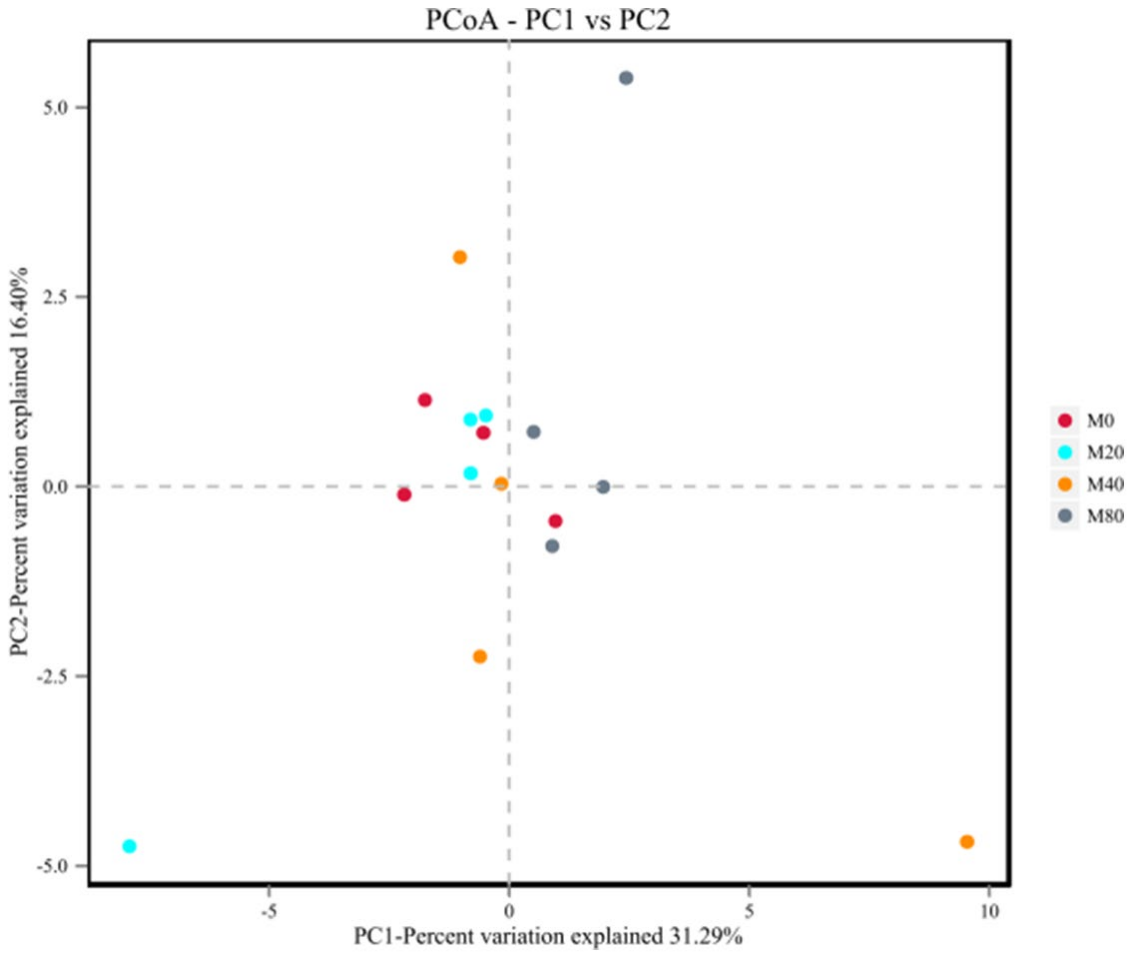
PCA plot of the microbiota of the cecum of broilers supplemented with melatonin.

Analysis of the microbial β diversity indices of the broiler cecum microorganisms in each group at the OTU classification level revealed that the contribution of principal component analysis (PCA) and the first principal component was 31.29%, and the contribution of the second principal component was 16.40% (Figure 3). The cecum microbial samples of groups M0 and M20 were centrally distributed, while those of groups M40 and M80 were relatively dispersed, suggesting that there were differences in the microbiota between them (*p* > 0.05).

### 3.3 Melatonin supplemented in the diet alters the relative abundance of the gut microbiota

**Figure 4:**
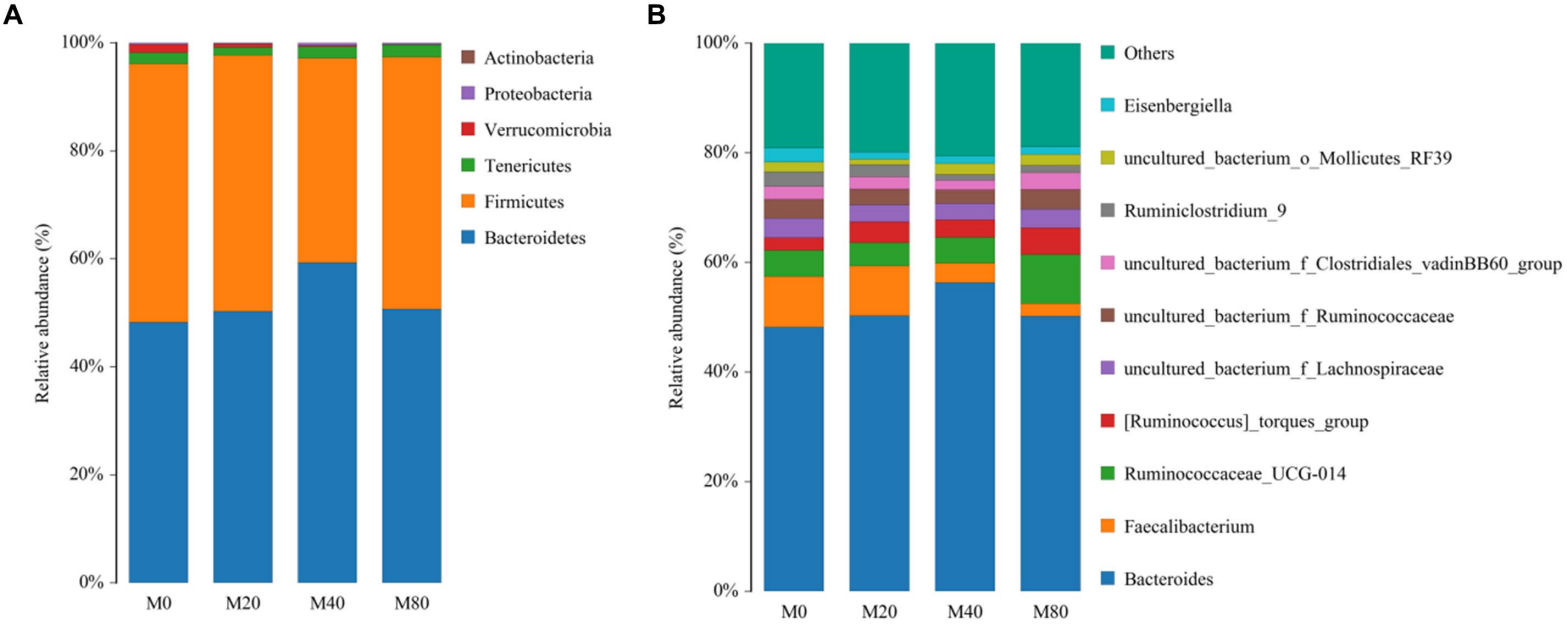
Microbiota composition of the cecum of broiler chickens. **(A)** Phylum level and **(B)** Genus.

At the phylum level, the top six phyla in terms of relative abundance were counted for all groups, and the dominant phyla of the intestinal contents in all groups were Bacteroidetes and Firmicutes (Figure 4A). The proportion of Bacteroidetes in the M20 (50.31%), M40 (59.28%), and M80 (50.69%) groups was greater than 50%. The relative abundance of the Bacteroides phylum in the intestinal contents of the M40 group appeared to be greater than that in the intestinal contents of the M0 group (48.25%). However, the relative abundance of the Firmicutes phylum in the intestinal contents of the M40 (37.84%) group was lower than that in the M0 (47.84%), M20 (47.39%), and M80 (46.70%) groups.

At the genus level, the relative abundances of the top 11 genera in Groups M0, M20, M40, and M80 were counted and compared to those in the other groups. The highest relative abundance of Bacteroides in the intestinal contents was detected in the M40 group (56.33%). The relative abundance of Faecalibacterium in the intestinal contents was greater in the M0 group (9.25%) than in the M20 (9.07%), M40 (3.55%), and M80 (2.23%) groups. The relative abundance of Ruminococcaceae in the intestinal contents of the M80 (8.98%) group was greater than that of the M0 (4.73%), M20 (4.26%), and M40 (4.69%) groups (Figure 4B).

### 3.4 Melatonin supplementation in the diet of broilers affects gut microbiota metabolic pathways and COGs

**Figure 5:**
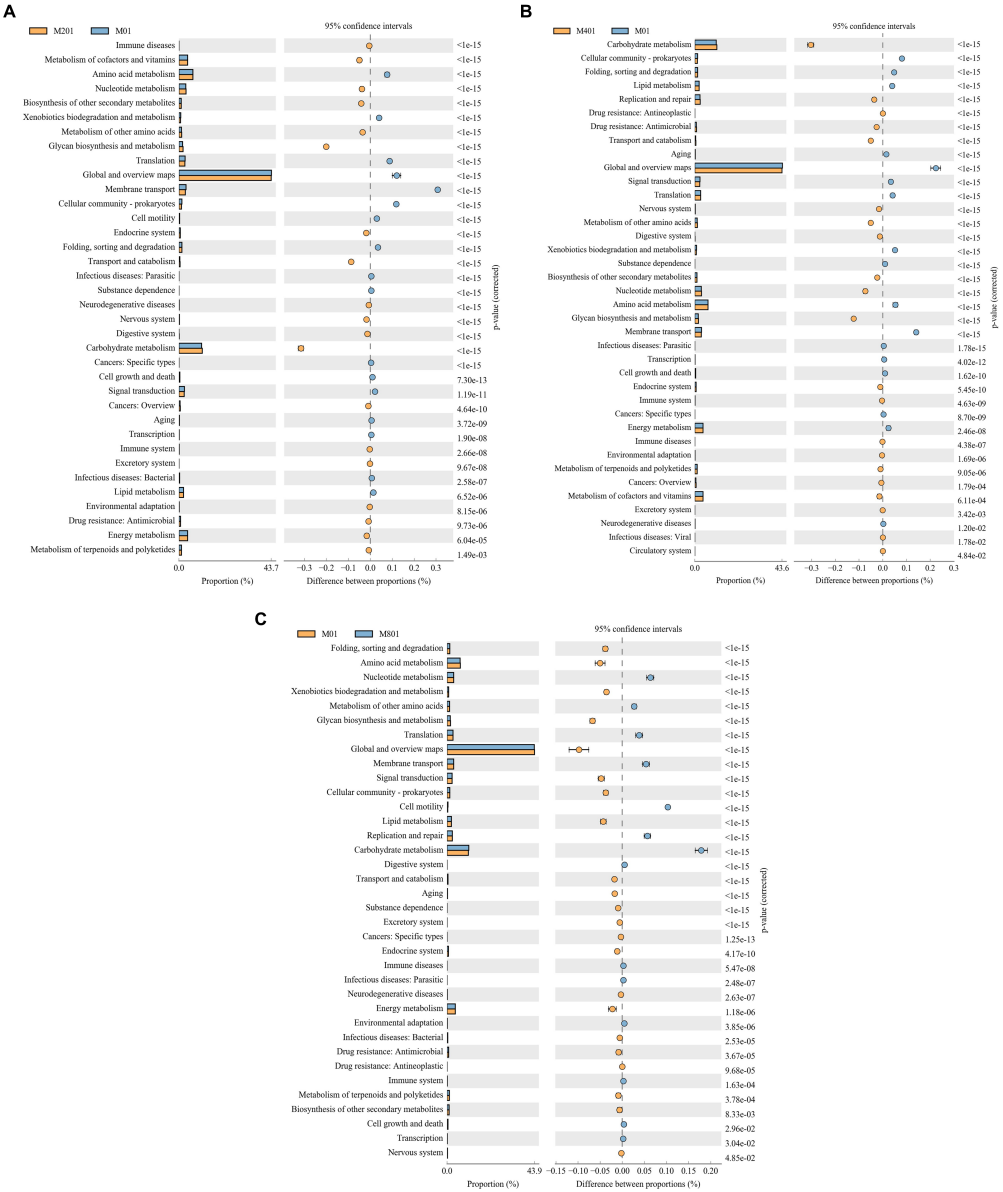
Differential analysis of KEGG metabolic pathways in cecal microorganisms of broilers supplemented with melatonin. **(A)** M20 group vs. M0 group; **(B)** M40 group vs. M0 group; and **(C)** M80 group vs. M0 group.

The functional gene composition of the samples was inferred using PICRUSt software by comparing species composition information obtained from the 16S sequencing data to analyze the differences in function between samples or groups. Differential analysis of KEGG metabolic pathways revealed the differences and changes in metabolic pathways of functional genes of microbial communities between samples of different groups, which is an effective means to investigate metabolic functional changes that occur in microbiota to adapt to environmental changes. Compared to those in the M0 group, immune diseases, metabolism of cofactors and vitamins, nucleotide metabolism, biosynthesis of other secondary metabolites, metabolism of other amino acids, glycan biosynthesis and metabolism, endocrine system, transport and catabolism, neurodegenerative diseases, nervous system, digestive system, carbohydrate metabolism, cancers: overview, immune system, excretory system, environmental adaptations, drug resistance: Antimicrobial, energy metabolism, and the metabolism of terpenoids and polyketides were significantly enriched pathways in the M20 group (*p* < 0.05) (Figure 5A). Carbohydrate metabolism, replication, and repair, drug resistance: Antineoplastic, drug resistance: Antimicrobial, transport and catabolism, nervous system, metabolism of other amino acids, digestive system, biosynthesis of other secondary metabolites, nucleotide metabolism, glycan biosynthesis and metabolism, endocrine system, immune system, immune diseases, environment adaptation, metabolism of terpenoids and polyketides, cancers: Overview, metabolism of cofactors and vitamins, excretory system, and the infectious diseases: Viral and circulatory system signaling pathways were significantly enriched pathways in the M40 group (*p* < 0.05) (Figure 5B). Nucleotide metabolism, metabolism of other amino acids, translation, membrane translocation, cell motility, replication and repair, carbohydrate metabolism, digestive system, immune disorders, infectious diseases: Parasitic, environmental adaptations, immune system, cell growth and death, and transcriptional pathways were significantly enriched pathways in the M80 group (*p* < 0.05) (Figure 5C). Among the metabolic pathways, nucleotide metabolism, metabolism of other amino acids, glucose metabolism, and metabolic signaling pathways of the digestive and immune systems were significantly enriched in the M20, M40, and M80 groups (*p* < 0.05).

**Figure 6:**
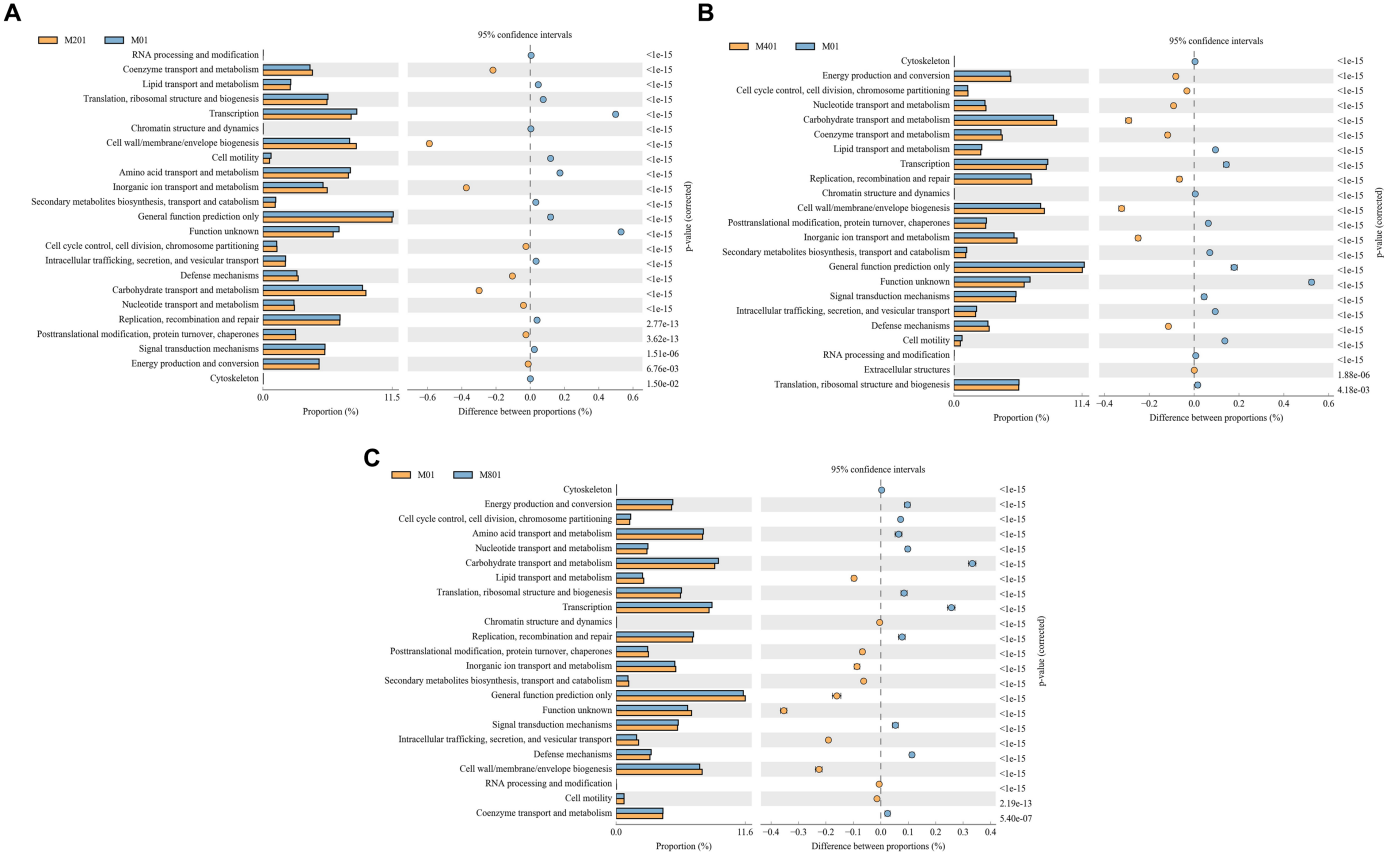
COG functional classification of cecal microbes in broilers supplemented with melatonin. **(A)** Histogram of COG metabolic pathways in the M20 group compared to the M0 group; **(B)** Histogram of COG metabolic pathways in the M40 group compared to the M0 group; and **(C)** Histogram of COG metabolic pathways in the M80 group compared to the M0 group.

The COG functional prediction analysis method is essentially the same as that used for KEGG and responds to the functional distribution and abundance of sequences in the sample. Coenzyme transport and metabolism, cell wall/membrane/envelope biogenesis, inorganic ion transport and metabolism, cell cycle control, cell division, chromosome partitioning, defense mechanisms, carbohydrate transport and metabolism, nucleotide transport and metabolism, post-translational modifications, protein turnover, chaperones, energy production, and conversion signaling pathways were significantly enriched in the M20 group compared to the M0 group (*p* < 0.05) (Figure 6A). Energy production and conversion, cell cycle control, cell division, chromosome partitioning, nucleotide transport and metabolism, carbohydrate transport and metabolism, coenzyme transport and metabolism, replication, recombination and repair, cell wall/membrane/envelope biogenesis, inorganic ion transport and metabolism, and defense mechanisms and extracellular structures pathways were significantly enriched in the M40 group (*p* < 0.05) (Figure 6B). The terms cytoskeleton, energy production and conversion, cell cycle control, cell division, chromosome partitioning, amino acid transport and metabolism, nucleotide transport and metabolism, carbohydrate transport and metabolism, translation, ribosomal structure and biogenesis, replication, recombination and repair, signal transduction mechanisms, defense mechanisms, and coenzyme transport and metabolism pathways were significantly enriched in the M80 group (*p* < 0.05) (Figure 6C). Among them, auxin transport and metabolism, cell wall/membrane/envelope biogenesis, carbohydrate transport and metabolism, and nucleotide transport and metabolism signaling pathways were significantly enriched in the M20, M40, and M80 groups (*p* < 0.05).

## 4 Discussion

A stable gut microbiota composition plays a crucial role in maintaining intestinal homeostasis in healthy individuals and positively regulates the brain–gut–microbiota axis through the microbiota (Chang et al., 2022). The cecum of broilers contains a large number of microorganisms, which leads to an increase in the incidence of intestinal inflammation and neuroinflammation and an increase in the number of pathogens if there is dysbiosis of the intestinal microbiota (de Theije et al., 2014). The intestinal microbiota may destabilize intestinal tight junction proteins to disrupt the intestinal integrity barrier and increase permeability; this process is known as a leaky gut (Carloni et al., 2021; Wang et al., 2023).

The ACE and Chao1 indices reflect the abundance of microbial communities, while the Simpson and Shannon indices represent the diversity of microorganisms (Zhang et al., 2021). Zhang B. et al. (2022) and Zhang L. et al. (2022) reported that the supplementation of mice with melatonin in the diet resulted in an increase in the number of OTUs and alleviated intestinal homeostatic dysregulation. In this study, the Venn diagram showed that the number of OTUs and the number of unique OTUs were greater in the 80 mg/kg melatonin treatment group than in the other groups, but according to the α diversity analysis, the addition of 80 mg/kg melatonin decreased the abundance of the cecal microbial community. This may be because the high concentrations of melatonin adjust the metabolism of the intestinal microbiota.

In the present study, at the phylum level, the core microbiota of the gut microorganisms in the groups were the Bacteroidetes and Firmicutes. Bacteroidetes is an important group of bacterial taxa that are mainly distributed in the distal intestine and provide energy to the host by fermenting indigestible polysaccharides, and SCFAs synthesized by these organisms can improve the host’s intestinal microecology and promote digestion and absorption (Putnam et al., 2022). Most members of the class Bacteroidetes have biofilms similar to those of Bacteroidetes, and these biofilms regulate the energy metabolism of the host and provide nutrients and energy to other microorganisms (Lapébie et al., 2019). The greater the ratio of the phylum Bacteroidetes is, the greater the body’s ability to absorb energy-related substances, while the smaller the ratio is, the more likely the animal is to become obese (Pan et al., 2023). The relative abundance of the Bacteroidetes phylum in broilers supplemented with 40 mg/kg of melatonin was greater than that in the other groups in the present study, which suggested that the addition of melatonin to the diet improved the ability of broilers to absorb nutrients and energy.

At the genus level, the core groups of gut microorganisms in the control group and the melatonin-supplemented groups at different doses were Bacteroides. Bacteroides are involved in intestinal carbohydrate and energy metabolism (Zafar and Saier, 2021) and are also key to immunomodulation, playing an important role in maintaining the stability of the immune system, and are involved in the synthesis of vitamin K, which is important for increasing bone density as well as preventing and treating osteoporosis (Walther et al., 2013). The relative abundance of Bacteroidetes was greater in the 40 mg/kg group in the present study than in the other groups of melatonin-supplemented broilers, which suggested that supplementation with 40 mg/kg melatonin is beneficial for maintaining intestinal homeostasis, regulating the immune system, and preventing osteoporosis. The data we previously published showed that adding melatonin to the diet impacted the body weight (live weight before slaughter) trend of broiler chickens. Among them, the M20 and M40 groups were heavier (Chen et al., 2023). In addition, compared with the control group, broiler chickens fed with 20 mg/kg (M20) of melatonin had a significantly higher average daily gain. While the average daily gain of the M20 group was not significantly higher than that of the M0 group. This change is not similar to the changes in microorganisms, but it is not entirely consistent. It may be more due to the involvement of melatonin in various metabolic processes in the body (Chen et al., 2023).

The relative abundance of Ruminococcaceae was greater in the melatonin-supplemented group than in the control group at the genus level. Ruminococcaceae are Gram-positive anaerobic bacteria belonging to the phylum Firmicutes (Ludwig et al., 2009) and are important gut microbial symbionts that degrade complex polysaccharides and convert them into a wide range of nutrients that can be utilized by the host. The genus Ruminococcaceae is effective in breaking down cellulose in the host’s digestive system, reducing the risk of colorectal cancer and decreasing diseases such as kidney stones (Juge, 2023). Clavijo and Flórez (2018) identified Clostridium spp., Ruminococcus, Lactobacillus spp., and Anaplasma spp. as the main genera of broiler intestinal microorganisms; however, the intestinal microbial diversity of chickens is relatively low compared to that of other animals (Rougière and Carré, 2010). Ruminococcus are ubiquitous in the gut, and their relative abundance has been linked to metabolism (Blanton et al., 2016). An increased relative abundance of Ruminococcus has an important role in stabilizing the intestinal barrier and reducing intestinal diseases as well as diarrhea (Juge, 2023). The relative abundance of Ruminococcus in broiler gut microorganisms also increased with the addition of melatonin, which improved the digestive function of broilers.

Liu et al. (2020) reported that melatonin highly regulates serum amino acid levels in mice with inflammatory bowel disease; moreover, melatonin can alleviate insulin resistance and obesity by activating the AMPKα/PPARα signaling pathway. The intestinal mucosa is the first line of defense against infection in the innate immune response. Modulation of the immune response prevents bacteria from penetrating the intestinal epithelial barrier (Carter et al., 2009). In this study, KEGG and COG enrichment analysis revealed that melatonin supplementation significantly affected the metabolic signaling pathways involved in nucleotide metabolism, metabolism of other amino acids, glucose metabolism, the digestive system, the immune system, auxin transport and metabolism, cell wall/membrane/envelope biogenesis, carbohydrate transport and metabolism, and nucleotide transport and metabolism in broiler chickens, which may be related to changes in the community structure of the intestinal microbiota. These enriched signaling pathways suggest that the addition of melatonin can boost metabolism, improve glucose metabolism balance, and protect the digestive system as well as the immune system.

## 5 Conclusion

The supplement of melatonin in the diet significantly increased the proportion of Bacteroidetes and Firmicutes phyla in the cecum, as well as the proportions of Bacteroides, Faecalibacterium, and Ruminococcaceae. It also affected nucleotide metabolism, amino acid metabolism, glucose metabolism, and digestive and immune system metabolic pathways, as well as coenzyme transport and metabolism, cell wall/membrane/envelope biogenesis, carbohydrate transport and metabolism, and nucleotide transport and metabolic pathways. Melatonin supplementation in broiler diets could improve the composition of the cecal microbiota, reduce the number of harmful microbiotas, and increase the number of beneficial microbiotas, thus regulating the cecal environment and promoting the health of broilers.

## Data Availability

The original contributions presented in the study are included in the article/supplementary material, further inquiries can be directed to the corresponding author.
